# Development and validation of the Chinese Acceptance Scale for university students (CAS) in Hong Kong, China

**DOI:** 10.3389/fpsyg.2026.1965526

**Published:** 2026-10-14

**Authors:** Daniel T. L. Shek, Liyang Luo, Chaoran Sun, Xiang Li, Lindan Tan, Esther Y. W. Shek

**Affiliations:** Department of Applied Social Sciences, The Hong Kong Polytechnic University, Kowloon, Hong Kong SAR, China

**Keywords:** acceptance, Chinese university students, diversity, measurement, scale, validation

## Abstract

**Background:**

This paper reports the development and validation of the Chinese Acceptance Scale (CAS) as a measure of acceptance of diversity among Chinese university students. Integrating Western and Chinese conceptions of acceptance of diversity, the CAS was initially conceptualized with four dimensions: “diversity of contact,” “comfort with difference,” “appreciation of others,” and “*kuan rong*” (tolerance of others).

**Methods:**

Study 1 involved content validation by an expert panel (*N* = 10), which evaluated 25 items and resulted in a 20-item instrument covering the four proposed dimensions. Study 2 examined the psychometric properties of the CAS using two waves of data from 963 university students, assessing factor structure, measurement invariance, convergent and discriminant validity, and reliability.

**Results:**

Confirmatory factor analysis provided support for the initial four-factor model. However, the results indicated that “appreciation of others” and “kuan rong” were better represented as a single factor. The revised three-factor model consisted of “diversity of contact,” “comfort with difference,” and “inclusive appreciation.” This model showed good factorial validity, measurement invariance over time, and satisfactory reliability. Convergent and discriminant validity were also supported at the total-scale level, although further examination of these properties at the subscale level is needed. Overall, the findings suggest that the CAS can be used to assess acceptance of diversity among Chinese university students.

## 1 Introduction

### 1.1 Conceptions of acceptance in the western literature

In the Western literature, acceptance of others is a multifaceted concept rooted in Philosophy, Social Work, and Psychology. As a pioneer, Wilhelm von Humboldt proposed the connection between accepting diversity and personal growth and maintained that embracing a “manifoldness of situations” is essential for holistic individual development (Von Humboldt, 2017). Kant’s influence extended this argument from the cognitive recognition of diversity to the principle of ontological equality. Since humans possess inherent dignity (*Würde*), the acknowledgement of equal rights and freedoms becomes indispensable for personal growth/education (*Bildung*). From this perspective, no one is inherently superior.

A related but distinct construct is experiential acceptance, which is regarded as “acknowledging and embracing experience” as an alternative to experiential avoidance (Chin and Hayes, 2017; Hayes, 2004; Mrazek et al., 2024). It is defined as “fundamental openness” and capacity to remain fully available to the present (Murrell and Kapadia, 2011; Williams and Lynn, 2010), comprising non-attachment, non-avoidance, non-judgment, tolerance, and willingness (Hall-Renn, 2007). This state requires embracing internal experiences exactly as they are (e.g., without judgment, evaluation, or distortion) and without attempting to suppress or cling to them (Berger, 1952; Cordova, 2001; MacDonald et al., 2010). Some related concepts of acceptance include self-acceptance (evaluation of oneself), acceptance of others (holding respect for other people), and acceptance of diversity which places particular emphasis on respecting people from different backgrounds and appreciating the differences involved (Arant et al., 2021; Chen and Hamilton, 2015; Emami and Safipour, 2013). Among them, acceptance of others and diversity occupies an important role in interpersonal relationships. This virtue is particularly important because there are findings suggesting that young people have become more egocentric across time (Twenge et al., 2008). In this paper, our focus is on acceptance of others, particularly diversity.

With reference to the tripartite view of attitude (i.e., behavior, affect and cognition), several domains of acceptance of diversity have been identified in the Western scientific literature. The first domain is one’s readiness to engage with people with different characteristics (i.e., “diversity of contact”) which is behavioral in nature. For example, the Diversity of Contact subscale of the Miville-Guzman Universality Diversity Scale Short Form (M-GUDS-S) (Miville et al., 1999) assesses interest in culturally diverse activities and willingness to engage with diverse others (Fuertes et al., 2000). A related behavioral emphasis appears in the Act domain of the Munroe Multicultural Attitude Scale Questionnaire (MASQUE), which assesses self-reported actions to challenge prejudice and facilitate communication across difference (Munroe and Pearson, 2006). Similarly, the Social Distance Scale assesses willingness to accept members of different groups in relationships of closeness, including friendship, workplace contact, and neighborhood proximity (Bogardus, 1933).

The second dimension is whether people feel comfortable interacting with people with different characteristics (i.e., “comfort of difference”; Fuertes et al., 2000; Miville et al., 1999). Accepting others means one has positive emotions toward others, at least not negative or rejecting emotions to others (i.e., “affective” dimension of acceptance). In terms of assessment, there are scales assessing affective dimension of acceptance. For example, the Comfort with Differences subscale of the M-GUDS-S evaluates individuals’ feelings of ease or discomfort when interacting with diverse others (Fuertes et al., 2000). Similarly, the Affective Subscale of the Multidimensional Attitudes Scale toward Persons with Disabilities (MAS) assesses emotional reactions to people with disabilities (Findler et al., 2007). The study of intergroup anxiety also provides relevant evidence. For example, Stephan and Stephan (1985) assessed emotional responses (e.g., awkwardness, self-consciousness, and confidence) when interacting with members of other racial or ethnic groups. These measures illustrate the assessment of emotional responses to interpersonal differences.

The third dimension is positive recognition of differences and the value of diverse perspectives (i.e., “appreciation of differences”; Hjerm et al., 2020; Walzer, 1997). It is consistent with the United Nations Educational, Scientific and Cultural Organization’s (1995) conceptualization of tolerance as involving respect, acceptance, and appreciation of diversity (United Nations Educational, Scientific and Cultural Organization, 1995, p. 2). This attribute can be regarded as the “cognitive” dimension of acceptance which has been assessed by the Relativistic Appreciation subscale of the M-GUDS-S (Fuertes et al., 2000) and the “know” domain of the MASQUE questionnaire, which addresses people’s awareness and understanding of cultural differences and social inequalities (Munroe and Pearson, 2006).

A concept closely related to acceptance is “tolerance” which is primarily cognitive in nature. Conceptually, tolerance can range from merely enduring differences to more positive forms of acceptance (Witenberg, 2019). Besides, Hjerm et al. (2020) suggested to understand tolerance as forbearance despite disagreement from a positive orientation toward diversity. Interestingly, inter-group toleration similarly involves accepting and accommodating differences even when individuals continue to object to particular out-group beliefs or practices (Verkuyten and Yogeeswaran, 2017) which has consequently been regarded as an important response to cultural and religious diversity in plural societies (Verkuyten et al., 2019).

To sum up, with reference to the tripartite conception of attitude, Western conceptions of acceptance cover not only willingness to engage with diverse others (i.e., behavioral acceptance) and feelings of emotional comfort in such social interactions (i.e., affective acceptance), but also appreciation and tolerance of difference (i.e., cognitive acceptance).

### 1.2 Conceptions of acceptance in Chinese literature

In traditional Chinese culture, acceptance-related concepts generally cover different objects, including the self, personal experiences, and others. Self-acceptance (“zi wo jie na”; 自我接纳) refers to an individual’s willingness to acknowledge and accept the actual self and its characteristics (Meng et al., 2017), including both positive and negative aspects of the self (Sun and Lu, 2017). Chinese scholars have discussed this orientation in relation to self-understanding, psychological adjustment, and personal development (Meng et al., 2017). At the interpersonal level, acceptance concerns responses to others whose beliefs or practices differ from one’s own. Drawing on Chinese thoughts, Wang (2019) discussed “toleration” in relation to inclusiveness and appreciation of cultural diversity.

Several related Chinese terms also cover different aspects of acceptance. “Jieshou” (接受) broadly refers to receiving or accepting something, whereas “jiena (接纳)” conveys a more psychologically oriented stance of acknowledgment, recognition, and voluntary acceptance (Meng et al., 2017; Ren et al., 2019). “Bao rong (包容)” emphasizes inclusiveness and openness to diversity and differing perspectives (Hong and Chao, 2012). “Kuan rong (宽容)” more specifically concerns tolerance toward behaviors, viewpoints, or differences that deviate from prevailing norms (Lu et al., 2017). In contrast, “kuan shu (宽恕)” refers more specifically to forgiveness following interpersonal wrongdoing or transgression (Li and Lu, 2018; Luo and Huang, 2004). Although these concepts overlap, they are not interchangeable.

With reference to Confucianism, acceptance is deeply embedded in the concept of “being strict with oneself and lenient to others” (“gong zi hou er bo ze yu ren”; 躬自厚而薄责于人). In addition, the Confucian ideal of harmony (he, 和) should not be equated with sameness (“tong”; 同). Li (2006) explains that harmony accommodates differences rather than requiring uniformity. This interpretation provides a philosophical basis for maintaining constructive relationships among people with differing perspectives. For Buddhism, it reframes experiential acceptance through deep insight into *dukkha* (suffering), *anicca* (impermanence), and *anatta* (non-self), recognizing that suffering and change are natural, thereby promoting non-judgmental, compassionate engagement with life that reduces ego-clinging and helps people accept life’s changes and recover from adversity (Somers, 2022). Buddhism also focuses on equality of all beings which suggests appreciation of others and “kuan rong.” For Taoism, it views the world as constantly changing and full of contradictory opposites, thus advocating the balancing and reconciliation of contradictions (Chai et al., 2024; Wang et al., 2022). More directly relevant to acceptance of diversity, Huang (2023) interprets “Zhuangzian respect” as extending not only to other persons but also to their different ways of life. This perspective goes beyond non-interference to include supporting others in pursuing their distinctive ways of life.

Several attributes can be identified in the Chinese conceptions of acceptance. First, acceptance involves tolerance and accommodation of differences rather than avoidance of those who differ from oneself (Chen, 2021). Second, it extends beyond mere tolerance to an appreciation of others and recognition of the value of diversity (Wang, 2019). Third, acceptance also has a relational orientation, emphasizing the maintenance of harmonious relationships despite differences. This commonality is reflected in the principles of “harmony without uniformity” (“he er bu tong”; 和而不同) and “seek common ground while reserving differences” (“qiu tong cun yi”; 求同存异) (Yao, 2023).

The above-mentioned attributes of acceptance are reflected in the Chinese concept of “kuan rong” which emphasizes tolerance and accommodation of differences while maintaining harmonious relationships, reflecting Chinese understanding of tolerance and harmonious coexistence (Chen, 2021; Li, 2006; Yao, 2023). In the Chinese context, the Interpersonal Tolerance scale of the Cross-Cultural (Chinese) Personality Assessment Inventory (Cheung et al., 2008) assesses the extent to which individuals tolerate people who are different from themselves, whereas the Interpersonal Tolerance Scale (IPTS) distinguishes warm tolerance, cold tolerance, and limits of tolerance in interpersonal contexts (Thomae et al., 2016). Building on the behavioral, affective, and cognitive aspects emphasized in Western research on acceptance, the initial CAS framework additionally incorporated “kuan rong” as Chinese philosophical accounts of kuan rong emphasize inclusion and accommodation of difference (Chen, 2021). It includes recognizing others’ entitlement to differ, managing discomfort or disagreement, and accommodating others rather than excluding them. Besides, “kuan rong” represents a culturally salient expression of acceptance that can involve non-calculation of different views, forgiveness, kindness, and taking others’ perspectives. Conceptually, this can be regarded to be primarily a cognitive dimension of acceptance. In fact, similar ideas of tolerating and accommodating differences are also evident in Western accounts (Hjerm et al., 2020; Verkuyten and Yogeeswaran, 2017).

### 1.3 Conceptual and methodological gaps in the literature

There are several conceptual and methodological gaps in the assessment of acceptance of diversity. First, extant research specifically assessing acceptance of diversity remains limited, particularly in the Chinese context. A targeted database search using the search term combination: title(acceptance) AND title(diversity) AND (title(scale) or title(assess*) or title(develop*)) was conducted in PsycINFO on September 10, 2026. The English record search only returned five records, none of which reported studies conducted in the Chinese context. It should be noted that PsycINFO is a comprehensive database covering published work in Social Sciences, including Psychology. Additionally, we conducted a complementary search of Chinese databases, including CSSCI and CNKI. As of September 10, there were only 4 citations when we used “acceptance” as the search term. Besides, no measure of acceptance of diversity has been comprehensively evaluated among Chinese university students in Hong Kong. In particular, only a small number of measures have addressed related constructs, including some isolated studies on inclusive leadership (Bao and Liao, 2024), forgiveness (Li and Yang, 2019), and tolerance (Zhang et al., 2022).

Second, empirical investigation of Chinese conceptions of acceptance is constrained by the lack of measures taking Chinese conceptions of acceptance (such as “kuan rong”) into account. While researchers can simply translate foreign scales into Chinese, translated scales often demonstrate poor psychometric properties (Morgado et al., 2014). Moreover, simple modifications are insufficient as they fail to capture individuals’ broader acceptance of diversity and overlook the culturally specific ways in which acceptance is constructed in Chinese culture (Cao et al., 2013). In fact, while Chinese perspectives emphasize appreciation and inclusiveness, accounts of harmony and interpersonal relatedness provide a relational context where these shared ideas are expressed (Wang, 2019; Yao, 2023). In this study, we integrated the behavioral, affective and cognitive attributes of acceptance highlighted in the Western literature with the concept of “kuan rong” in the Chinese literature in an integrated measure of acceptance, thus making it a conceptual advance.

Finally, there are few validated scales of acceptance of diversity in the field. Primarily, for scales suggesting multi-dimensional measurement of the construct, there is the need to ascertain the factor structure using confirmatory factor analysis. Although some studies have used confirmatory factor analysis (CFA) to examine related multidimensional measures (e.g., Hjerm et al., 2020; Kegel and DeBlaere, 2014), they did not assess factorial invariance across time. Furthermore, there are few studies on the measurement of convergent and discriminant validities of acceptance measures. Finally, there is no known study examining the factor structure, factorial invariance, convergent and discriminant validities, and reliability in a single study.

Against the above background, we conducted two studies to develop and validate a measure of acceptance of diversity that integrates relevant Western and Chinese conceptions of acceptance of diversity with reference to four dimensions: diversity of contact, comfort of contact, appreciation of others and “kuan rong.” In Study 1, we developed an initial item pool of 25 items and evaluated its content validity through expert review. In Study 2, we examined the factor structure of the 20-item CAS and refined the initial factor model based on confirmatory factor analysis (CFA) findings, and tested its factorial validity, measurement invariance, convergent validity, discriminant validity, and scale reliability.

## 2 Study 1: content and item validation study

### 2.1 Methodology

The initial four dimensions and item pool were developed through a theory-driven process integrating both Western and Chinese conceptions of acceptance of diversity described in the scientific literature. The initial items and dimensions of the CAS were developed by a team of experts in Social Work and Psychology with a mean of 15 years of experience. The initial conceptual framework distinguished four related dimensions based on existing literature: “diversity of contact” covers interacting with people from different backgrounds (e.g., Fuertes et al., 2000), “comfort with difference” covers ease in working and living with such people and positive feelings about harmonious coexistence (Miville et al., 1999), “appreciation of others” concerns recognizing others’ opinions, rights and worth despite mistakes or objectionable behavior inspiring by unconditional acceptance (David et al., 2013), and “kuan rong” concerns accommodating diversity and supporting inclusion (Caliskan and Saglam, 2012).

Consistent with previous studies (Leung and Shek, 2011; Schumm et al., 1986), content validity was assessed using expert ratings of item relevance and clarity, together with representativeness. A team of ten experts in Social Work and Psychology, with at leats 10 years of experience, evaluated the relevance, clarity, and representativeness of the proposed items using a 6-point rating scale. The item-level content validity index (I-CVI) expresses the proportion of experts endorsing an item on the relevant criterion. An I-CVI of 0.78 or above was treated as acceptable based on Zamanzadeh et al. (2015)‘s suggestions. Items falling below this criterion or receiving concerns from the experts were considered for revision or removal, consistent with established scale development protocols (Boateng et al., 2018).

### 2.2 Results and discussion

Table 1 reports I-CVI values, scale-level CVI summaries, and kappa values. The reported I-CVI values for relevance and clarity ranged from 0.80 to 1.00, with average S-CVI values of 0.94 and 0.95, respectively. Domain-representativeness values ranged from 0.90 to 1.00. Following experts’ suggestions, five items were removed due to inappropriate wording or expression. Following these suggestions, a content-validated 20-item instrument was eventually developed for validation in Study 2. The scale includes items on “diversity of contact” (Items 1, 5, 9, 13, and 17 assessing behavioral aspect of acceptance), “comfort with difference” (Items 2, 6, 10, 14, and 18 measuring affective aspect of acceptance), “appreciation of others” (Items 4, 8, 12, 16, and 20 primarily assessing cognitive-affective aspect of acceptance), and “kuan rong” (Items 3, 7, 11, 15, and 19 assessing primarily cognitive dimension of acceptance).

**Table 1:** Summary of content validity indexes (CVI) of the CAS.

Summary statistic	(1) Relevance		(2) Clarity	
	I-CVI	Multi-rater modified Kappa	I-CVI	Multi-rater modified Kappa
Range	0.80–1.00	0.79–1.00	0.80–1.00	0.79–1.00
% Of items meets standard (i.e., I-CVI = 0.78; Kappa = 0.75)	100%	100%	100%	100%
S-CVI (average)=	0.94		0.95	
S-CVI (proportion)=	0.94		0.95	
Average Kappa =	0.94		0.95	

(3) Representativeness				
Domains	I-CVI	Multi-rater modified Kappa		
1. Appreciation of others	1.00	1.00		
2. Diversity of contact	1.00	1.00		
3. Comfort with differences	0.90	0.90		
4. *Kuan rong*	0.90	0.90		
Range	0.90–1.00	0.90–1.00		
% Of items meets standard (i.e., I-CVI = 0.78; Kappa = 0.75)	100%	100%		
S-CVI (average)=	0.95			
S-CVI (proportion)=	0.93			
Average Kappa=	0.95			

In short, Study 1 provided preliminary evidence for the content validity of the CAS. Through expert review and CVI assessment, the initial item pool was refined from 25 items to a 20-item instrument. The retained items were considered relevant, clear, and culturally appropriate for assessing acceptance of diversity among Chinese university students. The resulting 20-item scale was then subjected to further validation in Study 2.

## 3 Study 2: psychometric properties of the CAS

Study 2 was conducted to test whether the proposed four-factor structure could be supported by the data. Besides factorial validity and longitudinal measurement invariance, convergent and discriminant validities, and reliability of the scale were examined. Specifically, we asked several research questions in this study.

Research question 1: Does the empirical evidence support the proposed four-factor structure of the CAS?

*Hypothesis 1a*: The proposed four-factor structure would be supported by acceptable model fit indices.

*Hypothesis 1b*: The related items of the CAS would significantly load on the initially proposed four-factor structure.

Research question 2: Are underlying dimensions of the CAS model longitudinally invariant?

*Hypothesis 2*: The CAS would be invariant over time.

Research question 3: Do the CAS whole scale and its subscales show convergent validity?

*Hypothesis 3a*: The correlation coefficients between the CAS measures and theoretically relevant measures, including the measures of other virtue scales, would be high and significant (*r* > 0.50) (Cohen et al., 2013).

*Hypothesis 3b*: The Average Variance Extracted (AVE) for the factors would be greater than 0.50 (Fornell and Larcker, 1981).

Research question 4: Do the CAS whole scale and its subscales show discriminant validity?

*Hypothesis 4a*: The CAS scales and subscales would show weak correlations with both theoretically irrelevant constructs and unrelated items (*r* < 0.30) (Cohen, 2013).

*Hypothesis 4b*: The square root of AVE for each factor would exceed the factor correlation coefficients with other latent factors.

*Hypothesis 4c*: The heterotrait-monotrait ratio (HTMT) would be lower than the 0.85 threshold (Henseler et al., 2015; Voorhees et al., 2016).

Research question 5: Are the CAS total scale and subscales reliable at Wave 1 and Wave 2?

*Hypothesis 5a*: The composite reliability (CR) of the whole scale and subscales would be higher than 0.70 (Hair et al., 1998).

*Hypothesis 5b*: Cronbach’s α and McDonald’s ω for the whole scale and subscales would be higher than 0.70 (Hayes and Coutts, 2020; Nunnally and Bernstein, 1994).

*Hypothesis 5c*: The mean inter-item correlations (MIIC) for the whole scale and the subscales would be higher than 0.30 across two waves (Robinson et al., 1991).

### 3.1 Methods

#### 3.1.1 Participants and procedures

Ethical approval was granted by the institutional review board of the corresponding authors’ institution. Through convenience sampling, student helpers recruited participants from eight public and two private universities in Hong Kong. Before completing the survey, participants’ informed consent was collected. Participants were informed that their participation was fully voluntary and that their responses would remain strictly confidential. To ensure data quality and retention rate, participants who completed at least 50% of the survey would receive a HK$100 supermarket voucher. Each participant was assigned a unique identifier so that their responses could be matched across two waves. Wave 1 (conducted from 1 July 2024 to 31 October 2024) yielded 1,973 eligible responses from 3,406 approached individuals. Wave 2 was administered around 1 year later (conducted from 1 July 2025 to 20 October 2025). Among the Wave 1 respondents, 963 participants completed more than 70% of the Wave 2 survey. Figure 1 presents the recruitment and retention flow. A two-wave longitudinal analytical sample (*N* = 963) was used to examine the factor structure of the CAS and its psychometric properties.

**Figure 1:**
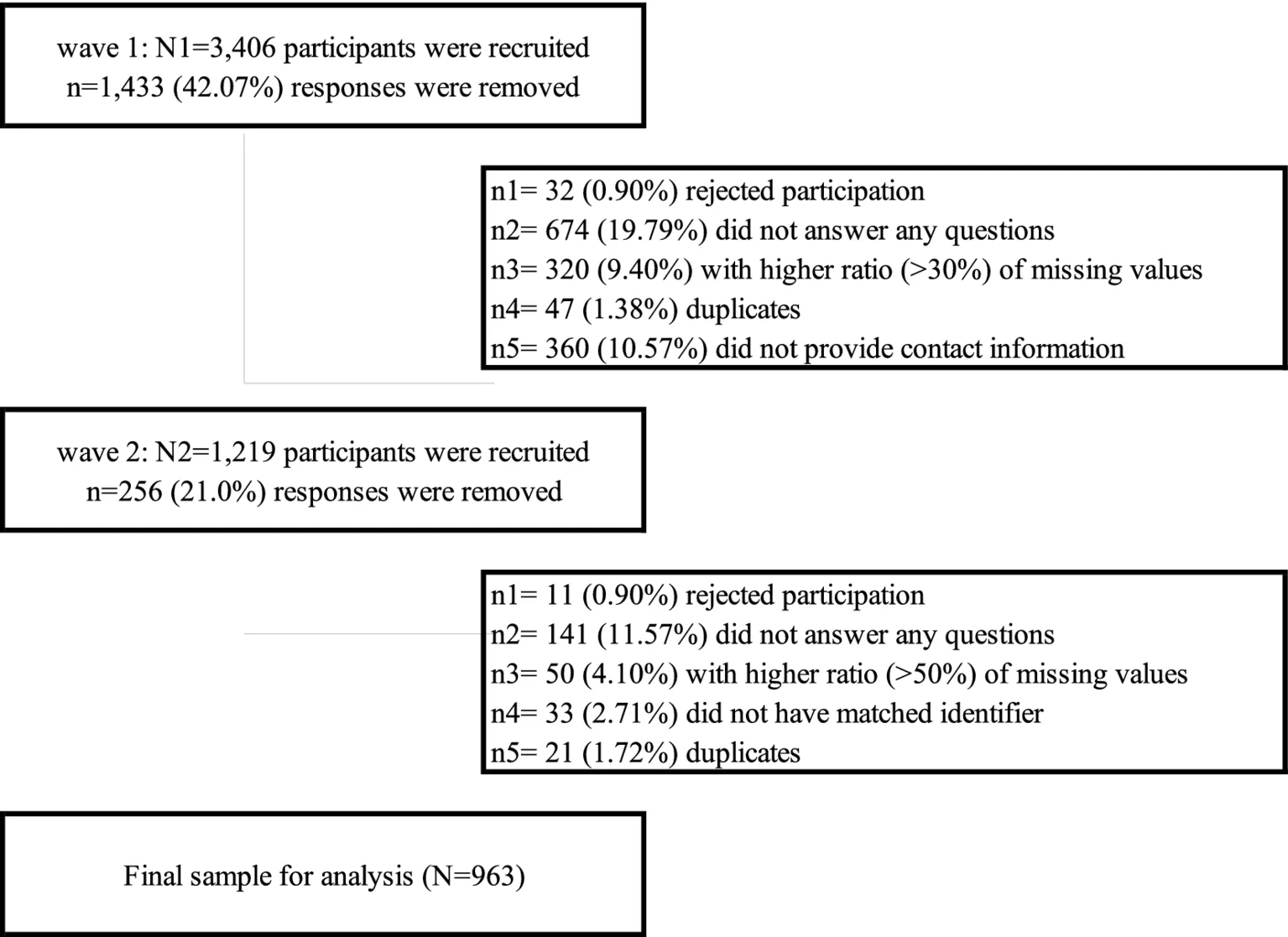
Data collection process.

#### 3.1.2 Measurements

Information on the demographic variables, covering age, gender, birthplace, education level of the parents, parental marriage status, family socioeconomic status, and grade, was collected. Participants responded to the CAS, which assessed their acceptance of diversity. Additionally, other virtue scales, including Respect, Care, Responsibility, and Integrity scales assessing students’ virtues, were included in this study (Shek et al., 2026). These virtue scales showed good content validity and reliability (Respect α_T1_ = 0.94, α_T2_ = 0.93; Care α_T1_ = 0.94, α_T2_ = 0.95; Responsibility α_T1_ = 0.93, α_T2_ = 0.93; Integrity α_T1_ = 0.92, α_T2_ = 0.93). These measures of virtues were used in examining the convergent validity of CAS.

Besides the virtue measures, Positive Relations with Others Scale (PRS; Ryff, 1989), Adolescent Responsible Behavior Scale (ARBS; Shek et al., 2021a; Zhou et al., 2021), Scale of Student Egocentrism (SSE; Shek et al., 2014), Student Moral Character Scale (SMCS; Shek et al., 2021b), and Moral Competence Scale (MCS; Shek and Zhu, 2019) were also utilized to assess convergent validity for the total acceptance of diversity scale and subscales. All scales demonstrated acceptable reliability (PRS α_T1_ = 0.77, α_T2_ = 0.76; ARBS α_T1_ = 0.88, α_T2_ = 0.89; SSE α_T1_ = 0.78, α_T2_ = 0.75; SMCS α_T1_ = 0.87, α_T2_ = 0.87; MCS α_T1_ = 0.70, α_T2_ = 0.76). Other than these scales, another item (i.e., “How do you rate your morality?”) was used to assess the convergent validity of CAS. Finally, the Prosocial Attitude Scale (PAS; adapted from the Chinese Positive Youth Development Scale; Shek et al., 2007) was used to examine the convergent validity of CAS.

Four conceptually uncorrelated indicators were included in the discriminant validity test (Schumm et al., 1986; Shek et al., 1993), including (1) Do you like Korean movies? (2) Do you like playing basketball? (3) Do you agree that Hong Kong has a good climate? and (4) Do you like ice cream? Participants’ responses were assessed on a 4-point scale (1 = Strongly dislike, 4 = Strongly like). The use of conceptually unrelated indicators has precedents in scale-validation research (Schumm et al., 1986).

#### 3.1.3 Statistical analysis

Descriptive statistics and Pearson correlations were computed using IBM SPSS Statistics for Windows (Version 30.0) (IBM Corp, 2024). The assumption of data normality was checked to ensure prerequisites for CFA were met. Data normality was defined as absolute skewness ≤2.0 and absolute kurtosis ≤7.0 (Curran et al., 1996; Kline, 2023). CFA and measurement invariance tests were conducted using SEM in R 4.3.3 to test whether the initial scale structure could be supported by the data and to examine the CAS’s factorial validity, convergent validity, discriminant validity, reliability, and temporal stability. Parameters were estimated via robust maximum likelihood (RML), which reduces standard errors biased by multivariate non-normality. Missing values were handled using “full-information maximum likelihood” (FIML). As we have a clear *a priori* conceptual model, CFA was selected to test the specified four-dimensional model (Bollen, 1989; Lambert and Newman, 2023; Shek et al., 2026).

To test Hypotheses 1a and 1b, factorial validity was assessed via CFA to determine whether observed items reflected the CAS’s latent factor structure. Data from both waves were combined into a single model, treating time as a grouping variable, yielding one set of fit indices to evaluate factor structure stability over time. According to Brown (2015), the model fits the data adequately when “the root-mean-square error of approximation” (RMSEA) and the “standardized root-mean-square residual” (SRMR) are lower than 0.10 and 0.08, respectively (Hu and Bentler, 1999). Besides, the values of the “Comparative Fit Index” (CFI) and the “Tucker-Lewis Index” (TLI) are greater than 0.90 (Bentler and Bonett, 1980; Hoyle, 1995). For measurement invariance (Hypothesis 2), configural, metric, scalar, and strict models were compared, grouping by time. |ΔCFI| and |ΔRMSEA| were used to assess invariance (Vandenberg and Lance, 2000) as χ^2^ difference tests are overly sensitive in large samples when *N* > 300, often rejecting nested models despite trivial differences (Cheung and Rensvold, 2002). For metric invariance, acceptable thresholds are “|ΔCFI| <0.010, |ΔRMSEA| <0.015, and |ΔSRMR| <0.030” (Chen, 2007). For scalar and strict invariance models, the acceptable thresholds are “|ΔCFI| <0.010 and |ΔRMSEA| <0.015, with |ΔSRMR| <0.010” (Chen, 2007).

#### 3.1.4 Attrition analysis

There was no significant group difference between stayers (*N* = 963) and leavers (*N* = 1,008) in CAS total scores (*t* (1,969) = −0.96, *p* = 0.34), low-income social welfare (*χ*^2^ (2) = 0.59, *p* = 0.75), parents’ marital status (*χ*^2^ (4) = 1.45, *p* = 0.84), father’s highest education (*χ*^2^ (7) = 6.35, *p* = 0.50) and mother’s highest education (*χ*^2^ (7) = 5.76, *p* = 0.57). Participants were less likely to respond in Wave 2 if they were male (*χ*^2^ (1) = 8.04, *p* < 0.01), born in Hong Kong (*χ*^2^ (3) = 8.79, *p* < 0.05), or were younger (*t* (1,866.24) = −2.33, *p* < 0.05).

### 3.2 Results

#### 3.2.1 Descriptive statistics

Means, standard deviations, skewness, and kurtosis were computed for each item of the student acceptance scale. All items were approximately normally distributed, with absolute univariate skewness values between 0.26 and 1.08 and kurtosis values between 0.01 and 2.29, both within the commonly accepted thresholds of 2 for skewness and 7 for kurtosis for the SEM assumption check. The robust maximum likelihood (RML) estimation method was therefore used in subsequent analyses because of the violation of multivariate normality. Demographic details are in Table 2, and item descriptive statistics are in Table 3.

**Table 2:** Sociodemographic descriptive statistics (*N* = 963).

Sociodemographic variables		*n*	*%*
Gender	Female	694	72.4
	Male	265	27.6
Age	18 or lower	21	2.2
	19 to 24	861	90.3
	25 or above	71	7.5
Birth place	Local	601	62.8
	Non-local	356	36.9
Father’s highest education level	Associate degree, bachelor’s, or above	377	39.2
	Secondary education	394	40.9
	Primary school or less	123	12.8
	Not sure	68	7.1
Mother’s highest education level	Associate degree, bachelor’s, or above	331	35
	Secondary education	412	43.4
	Primary school or less	146	15.3
	Not sure	59	6.2
Parent’s marital status	Married	789	82.4
	Not married	168	17.5
Being categorized as low-income family	No	907	94.4
	Yes	23	2.4
	Not sure	31	3.2
Grade	Sophomore	336	35.1
	Junior	289	30.2
	Senior	332	34.7

**Table 3:** Mean, standard deviation, skewness, and kurtosis of items over two waves.

Item	Wave 1				Wave 2			
	*M*	*SD*	Skewness	Kurtosis	*M*	*SD*	Skewness	Kurtosis
1.我會去嘗試了解這個世界上的多元文化	4.88	0.92	−0.71	0.95	4.96	0.86	−0.72	1.05
I will try to understand the diverse cultures of this world (DC).								
2.我與不同背景的人待在一起時, 感覺很自然	4.83	0.94	−0.56	0.17	4.90	0.80	−0.32	0.01
I feel comfortable interacting with people from different backgrounds (CD).								
3.我贊同社會受益於多樣化的傳統和生活方式	4.27	1.13	−0.26	−0.48	4.97	0.92	−1.05	1.98
I believe society benefits from diverse traditions and lifestyles (IA).								
4.我尊重不同背景人士的意見	4.24	1.12	−0.27	−0.43	4.96	0.83	−0.50	0.24
I respect the opinions of individuals from different backgrounds (IA).								
5.我會去主動認識生活在這個城市的其他族群的習俗	4.79	0.96	−0.72	0.95	4.65	1.00	−0.58	0.45
I will actively learn about the customs of other ethnic groups living in this city (DC).								
6. 我樂意與不同背景的人士合作	4.80	0.88	−0.43	0.25	4.63	0.99	−0.49	0.12
I am comfortable working with people from diverse backgrounds (CD).								
7.我們應該主動幫助不同背景的同學融入校園	4.91	0.91	−0.74	1.05	4.94	0.91	−0.70	0.54
We should proactively help classmates from diverse backgrounds integrate into campus life (IA).								
8.我尊重不同背景人士的權利	4.86	0.85	−0.61	0.88	4.93	0.88	−0.78	1.33
I respect the rights of people from different backgrounds (IA).								
9.我會參加能夠認識不同背景人士的社交活動	4.25	1.25	−0.45	−0.36	4.95	0.89	−0.84	1.36
I will participate in social activities that allow me to meet people from diverse backgrounds (DC).								
10.我與不同背景的人相處融洽	4.14	1.25	−0.37	−0.40	4.92	0.85	−0.65	0.75
I get along well with people from different backgrounds (CD).								
11.我們應該以寬容的態度與不同背景的人士相處	4.72	0.95	−0.47	0.19	4.61	0.96	−0.48	0.42
We should interact with people from different backgrounds with tolerance (IA).								
12.我認同不同背景人士的能力	4.63	0.94	−0.48	0.35	4.56	0.92	−0.46	0.51
I recognize the abilities of people from diverse backgrounds (IA).								
13.我會與不同背景的人士溝通交談	4.70	0.99	−0.64	0.67	4.26	1.25	−0.56	−0.15
I engage in communication with people from different backgrounds (DC).								
14.我樂意與不同背景的人居住在同一個城市	4.67	0.95	−0.62	0.98	4.17	1.24	−0.45	−0.24
I am happy to live in the same city with people from diverse backgrounds (CD).								
15.我願意接納不同背景人士	4.97	0.89	−0.81	1.04	5.02	0.89	−1.08	2.29
I am willing to accept people from different backgrounds (IA).								
16.即使一個人犯了一些錯誤, 我仍然會給予他/她 應有的尊重	4.96	0.85	−0.53	0.35	4.97	0.84	−0.61	0.53
Even if someone makes mistakes, I will still give him/her the respect he/she deserves (IA).								
17.我會參與不同背景的團體所組織的活動	4.28	1.29	−0.59	−0.22	4.69	0.99	−0.65	0.85
I participate in activities organized by groups from diverse backgrounds (DC).								
18.我樂意看到不同背景的人在這城市中和諧共處	4.24	1.28	−0.52	−0.30	4.62	1.00	−0.53	0.49
I am happy to see people from different backgrounds living harmoniously in my city (CD).								
19.我們應該主動了解不同背景的人的需要, 以協助他們融入社會	4.59	0.96	−0.43	0.31	4.68	1.07	−0.71	0.58
We should proactively understand the needs of people from different backgrounds to help them integrate into society (IA).								
20.無論一個人的行為如何, 他/她仍有值得欣賞的地方	4.61	0.90	−0.26	−0.02	4.63	1.12	−0.84	0.79
Regardless of how a person behaves, there is still something about him or her that deserves appreciation (IA).								

DC, Diversity of contact; CD, Comfort with difference; IA, Inclusive appreciation.

#### 3.2.2 Factor structure of CAS

CFA was conducted to examine the initially proposed four-factor structure (Table 4). Regarding model fit, although some goodness of fit measures were marginally acceptable (RMSEA = 0.10, SRMR = 0.06), CFI and TLI values were not high. Hence, the findings provided partial support for Hypothesis 1a only. Nevertheless, the findings provided support for Hypothesis 1b because all related items of CAS loaded significantly on the proposed four-factor structure. However, correlations among AO, CD, and KR were extremely high (*r* = 0.97–1.00), producing a non–positive definite latent covariance matrix. These findings suggested that the dimensions may represent a highly similar underlying construct (Comrey and Lee, 1992). To address this issue, we decided to combine some conceptually redundant factors, balancing statistical robustness with theoretical coherence. Additional analyses showed that the original KR and AO dimensions could be synthesized into a single factor which could be named “inclusive appreciation (IA)” dimension. Conceptually, while DC and CD reflect the behavioral and affective aspects of acceptance, respectively, IA represents the cognitive attribute of diversity, merging the traditional KR with AO. The finalized three-dimensional 20-item instrument, refined through expert consultation and grounded in theory, is presented in Table 3.

**Table 4:** Standardized factor loading and factor correlation from CFA of the CAS (four-factor based on content validation).

Standardized factor loadings (T1)			Correlation between factors (T1)				
Diversity of contact	Item 1	0.66		DC	CD	KR	AO
	Item 5	0.72	DC		0.93	0.82	0.75
	Item 9	0.79	CD			1.00	0.97
	Item 13	0.78	KR				1.00
	Item 17	0.72	AO				
Comfort with difference	Item 2	0.64					
	Item 6	0.73					
	Item 10	0.73					
	Item 14	0.72					
	Item 18	0.68					
Kuan rong	Item 3	0.67	Correlation between factors (T2)				
	Item 7	0.76		DC	CD	KR	AO
	Item 11	0.79	DC		0.96	0.85	0.76
	Item 15	0.79	CD			1.00	0.94
	Item 19	0.70	KR				1.00
Appreciation of others	Item 4	0.73	AO				
	Item 8	0.79					
	Item 12	0.77					
	Item 16	0.58					
	Item 20	0.46					

All the loadings were significant at the 0.001 level. DC, Diversity of contact; CD, Comfort with difference; KR, kuan rong; AO, Appreciation of others.

To validate the factor structure and compare the model fit indices of the CAS against other alternative models, CFA was conducted using the full two-wave sample. Model fit was compared across a one-factor model, the newly refined three-factor model, the original four-factor model based on content validation, and a three-factor orthogonal model. The three-factor orthogonal model retained the three latent factors but the covariance among them were constrained to zero, thereby testing whether these three factors could be treated as independent.

Longitudinal CFA across two waves indicated that the refined three-factor model showed acceptable model fit (*χ*^2^ (328) = 3501.52, *p* < 0.001; CFI = 0.90, TLI = 0.88, RMSEA = 0.085, and SRMR = 0.056). Model comparison findings revealed that the refined three-factor model showed better CFI, TLI, RMSEA, SRMR, and *χ*^2^ values than the one-factor model and the orthogonal model (Table 5). Standardized factor loadings ranged from 0.52 to 0.77 (Table 6), and every observed variable loaded significantly on the latent factors (*p* < 0.001). Longitudinal CFA indicated that the three-factor model provided a better fit to the data than the originally hypothesized four-factor model. Compared with the originally hypothesized four-factor model, the three-factor model had the same degrees of freedom (*df* = 328) but yielded a lower *χ*^2^ (*χ*^2^ = 3501.52), Akaike Information Criterion (AIC) and Bayesian Information Criterion (BIC) values (see Table 5). Given the principle of parsimony and these information criteria, the three-factor structure was retained for further analysis.

**Table 5:** Longitudinal CFA model comparisons.

Model	*χ^2^*	*df*	*χ^2^*/*df*	CFI	TLI	RMSEA	SRMR	AIC	BIC
One-factor model	4279.579	338	12.661	0.827	0.806	0.109	0.065	85279.433	85953.890
Three-factor model	3501.517	328	10.675	0.900	0.883	0.085	0.056	86926.146	87682.741
Three-factor orthogonal model	7625.716	334	22.831	0.671	0.626	0.151	0.336	88633.57	89330.14
Four-factor model	3562.735	328	10.862	0.859	0.836	0.101	0.061	87722.707	88457.049

**Table 6:** Standardized factor loading and factor correlation from CFA of the CAS (three-factor).

Standardized factor loadings (T1)			Correlation and factor AVE values on the diagonal (T1)			
Diversity of Contact	Item 1	0.61		DC	CD	IA
	Item 5	0.71	DC	0.52	0.95	0.81
	Item 9	0.76	CD		0.46	0.98
	Item 13	0.74	IA			0.46
	Item 17	0.74				
Comfort with difference	Item 2	0.62				
	Item 6	0.71				
	Item 10	0.72	Correlation and factor AVE values on the diagonal (T2)			
	Item 14	0.68		DC	CD	IA
	Item 18	0.65	DC	0.54	0.96	0.82
Inclusive Appreciation	Item 3	0.66	CD		0.49	0.97
	Item 4	0.71	IA			0.48
	Item 7	0.67				
	Item 8	0.73				
	Item 11	0.75	HTMT		T1	T2
	Item 12	0.69	DC-CD		0.95	0.97
	Item 15	0.77	DC-IA		0.82	0.82
	Item 16	0.58	CD-IA		0.99	0.97
	Item 19	0.70				
	Item 20	0.52				

Kuan rong and appreciation of others were combined into inclusive appreciation. All the loadings were significant at the 0.001 level. DC, Diversity of contact; CD, Comfort with difference; IA, Inclusive appreciation.

Latent factor correlations ranged from 0.81 to 0.98 (Table 6). The path diagram of the CFA model is shown in Figure 2. To account for item-level dependence arising from overlapping wording or content, five pairs of residual covariances were specified based on modification indices and the researchers’ substantive review of item wording. Items 14 and 15 are related to acceptance of people from different backgrounds (MI = 168.61). Items 14 and 18 share the phrase “I am happy to” with reference to positive affect toward living with or witnessing harmonious coexistence among people from different backgrounds in the same city (MI = 59.66). Item 3 and item 4 both capture positive evaluation of diverse traditions, lifestyles, and opinions (MI = 135.3). Items 9 and 18 refer to social/public context involving people from different backgrounds (MI = 81.75). Items 16 and 20 both focus on appreciating a person regardless of his/her previous mistakes or behaviors (MI = 121.54). These residual covariances were specified only when a high modification index for a given item pair was accompanied by substantive overlap in item content or similar wording.

**Figure 2:**
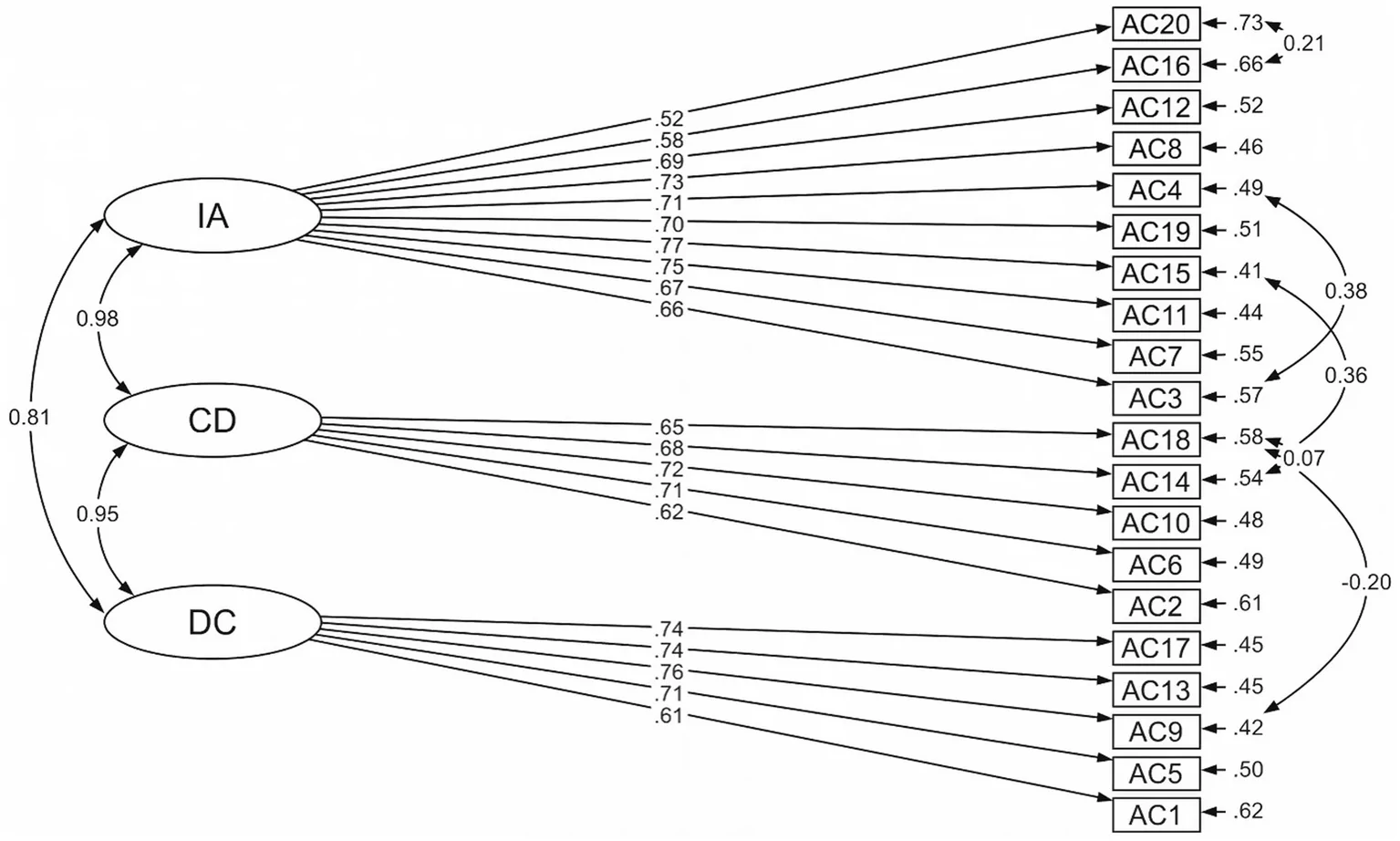
Model diagram of CFA with standardized pathway estimates (Time 1). IA, Inclusive appreciation; CD, Comfort with difference; DC, Diversity of contact.

#### 3.2.3 Measurement invariance

To test whether the factor structure of the CAS was stable across time, longitudinal confirmatory factor analysis was used to assess measurement invariance across waves (Table 7). First, a configural model was tested, allowing all parameters to be freely estimated across time, and showed an overall acceptable fit, χ^2^(324) = 2758.18, *p* < 0.001; CFI = 0.90; TLI = 0.88; RMSEA = 0.085; SRMR = 0.056. Second, a metric model was tested by constraining factor loadings to be equal across time. Third, scalar invariance was examined by additionally constraining item intercepts to be equal. Finally, factor loadings, intercepts, and residual errors were constrained across time to test strict invariance.

**Table 7:** Invariance of CAS across two waves with time as grouping.

Model	*χ* ^2^	*df*	CFI	TLI	RMSEA [90%CI]	SRMR	$|\Delta \chi^{2}|$	*p*	|ΔCFI|	|ΔRMSEA|	|ΔSRMR|
Configural	2758.18	324	0.900	0.883	0.085 [0.082, 0.089]	0.056					
Metric	2776.43	341	0.900	0.889	0.083 [0.080, 0.087]	0.058	15.33	0.57	0.000	0.002	0.002
Scalar	2797.94	358	0.900	0.894	0.081 [0.078, 0.085]	0.058	21.51	0.20	0.000	0.002	0.000
Strict	2953.56	378	0.895	0.894	0.081 [0.078, 0.085]	0.059	84.91	<0.001	0.005	0.000	0.001

Results suggested acceptable model fit across longitudinal invariance models. Across models from configural to strict invariance, changes in approximate fit indices (|ΔCFI| = [0.000, 0.005], |ΔRMSEA| = [0.000, 0.002], and |ΔSRMR| = [0.000, 0.002]) remained within invariance criteria proposed by Chen (2007). These results support the configural, metric, scalar, and strict invariance across two waves. Although the strict model showed a significant *χ*^2^ difference compared with the scalar model (Δ*χ*^2^ = 84.91, *p* < 0.001), this is not problematic because the *χ*^2^difference test is sensitive to large sample sizes. Therefore, based on the changes in the approximate fit indices, Hypothesis 2 was supported.

#### 3.2.4 Convergent validity

Regarding convergent validity, the total score of the acceptance of the CAS significantly correlated with other virtue scales, including Respect ( ∣r∣∈[0.33,0.65],p<0.01 ), Care ( ∣r∣∈[0.41,0.67],p<0.01 ), Responsibility ( ∣r∣∈[0.33,0.61],p<0.01 ), and Integrity Scales ( ∣r∣∈[0.32,0.60],p<0.01 ) (Table 8). Furthermore, the total score of the CAS also significantly correlated with the PRS ( ∣r∣∈[0.33,0.46],p<0.01 ), ARBS ( ∣r∣∈[0.34,0.52],p<0.01 ), SSE ( ∣r∣∈[0,0.05],p<0.01 ), SMCS ( ∣r∣∈[0.27,0.48],p<0.01 ), MCS ( ∣r∣∈[0.29,0.39],p<0.01 ), and PAS items ( ∣r∣∈[0.12,0.39],p<0.01 ).

**Table 8:** Correlation between the CAS and subscales with other virtue scale for convergent validity.

Correlation	Respect	Respect	Caring	Caring	Responsibility	Responsibility	Integrity	Integrity	PRS	PRS	ARBS	ARBS
	T1	T2	T1	T2	T1	T2	T1	T2	T1	T2	T1	T2
DC	0.48^**^	0.32^**^	0.57^**^	0.37^**^	0.49^**^	0.30^**^	0.45^**^	0.28^**^	0.37^**^	0.33^**^	0.49^**^	0.34^**^
T1												
DC	0.27^**^	0.41^**^	0.37^**^	0.54^**^	0.28^**^	0.52^**^	0.25^**^	0.41^**^	0.36^**^	0.45^**^	0.36^**^	0.45^**^
T2												
CD	0.56^**^	0.34^**^	0.61^**^	0.37^**^	0.55^**^	0.31^**^	0.55^**^	0.36^**^	0.37^**^	0.30^**^	0.47^**^	0.31^**^
T1												
CD	0.29^**^	0.48^**^	0.36^**^	0.56^**^	0.27^**^	0.52^**^	0.28^**^	0.49^**^	0.30^**^	0.41^**^	0.30^**^	0.41^**^
T2												
IA	0.69^**^	0.41^**^	0.68^**^	0.39^**^	0.65^**^	0.37^**^	0.63^**^	0.41^**^	0.49^**^	0.35^**^	0.50^**^	0.32^**^
T1												
IA	0.37^**^	0.61^**^	0.42^**^	0.66^**^	0.35^**^	0.62^**^	0.37^**^	0.59^**^	0.35^**^	0.41^**^	0.35^**^	0.46^**^
T2												
Acceptance total	0.65^**^	0.39^**^	0.67^**^	0.42^**^	0.61^**^	0.36^**^	0.60^**^	0.39^**^	0.44^**^	0.35^**^	0.52^**^	0.34^**^
T1												
Acceptance total	0.33^**^	0.56^**^	0.41^**^	0.64^**^	0.33^**^	0.61^**^	0.32^**^	0.55^**^	0.33^**^	0.46^**^	0.35^**^	0.47^**^
T2												

^*^p < 0.01, ^**^p < 0.001. PRS, Positive Relations with Others Scale, ARBS, Adolescent Responsible Behavior Scale.

In Tables 8 and 9, CAS subscales significantly correlated with the PRS ( ∣r∣∈[0.30,0.49],p<0.01 ), the ARBS ( ∣r∣∈[0.30,0.50],p<0.01 ), SSE ( ∣r∣∈[0,0.11],p<0.01 ), SMCS ( ∣r∣∈[0.26,0.50],p<0.01 ), MCS ( ∣r∣∈[0.24,0.44],p<0.01 ), morality ( ∣r∣∈[0.17,0.28],p<0.01 ) and PAS items ( ∣r∣∈[0.06,0.40],p<0.01 ). Additionally, AVE values of the three CAS factors ranged from 0.46 to 0.54. Specifically, the AVE value of DC is greater than 0.50 across two waves, while the AVE values for CD and IA fell slightly below the 0.50 threshold. Therefore, Hypothesis 3a was supported, and Hypothesis 3b was partially supported (see Tables 6, 9). Accordingly, while convergent validity of the CAS whole scale was demonstrated, the evidence for the convergent validity of the CAS subscales was not strong.

**Table 9:** Correlation between the CAS and subscales with other convergent scales and items.

Measure/item	DC(T1)	DC(T2)	CD(T1)	CD(T2)	IA(T1)	IA(T2)	Acceptance Total (T1)	Acceptance Total (T2)
SSE (T1)	0.04	0.03	−0.00	−0.03	−0.07^*^	−0.09^**^	−0.03	−0.05
SSE (T2)	0.02	0.08^*^	−0.04	0.02	−0.11^**^	−0.05	−0.05	−0.00
SMCS (T1)	0.42^**^	0.26^**^	0.44^**^	0.26^**^	0.50^**^	0.29^**^	0.48^**^	0.27^**^
SMCS (T2)	0.30^**^	0.36^**^	0.28^**^	0.36^**^	0.31^**^	0.45^**^	0.33^**^	0.42^**^
MCS (T1)	0.28^**^	0.24^**^	0.31^**^	0.25^**^	0.38^**^	0.31^**^	0.35^**^	0.29^**^
MCS (T2)	0.24^**^	0.30^**^	0.29^**^	0.36^**^	0.32^**^	0.44^**^	0.30^**^	0.39^**^
(1) (T1)	0.21^**^	0.19^**^	0.20^**^	0.18^**^	0.24^**^	0.28^**^	0.23^**^	0.22^**^
(1) (T2)	0.17^**^	0.17^**^	0.21^**^	0.20^**^	0.22^**^	0.26^**^	0.21^**^	0.24^**^
(2) (T1)	0.35^**^	0.29^**^	0.30^**^	0.24^**^	0.36^**^	0.30^**^	0.36^**^	0.30^**^
(2) (T2)	0.30^**^	0.37^**^	0.25^**^	0.32^**^	0.28^**^	0.40^**^	0.30^**^	0.39^**^
(3) (T1)	0.40^**^	0.33^**^	0.31^**^	0.26^**^	0.39^**^	0.33^**^	0.39^**^	0.32^**^
(3) (T2)	0.34^**^	0.40^**^	0.27^**^	0.33^**^	0.28^**^	0.37^**^	0.31^**^	0.39^**^
(4) (T1)	0.12^**^	0.06^**^	0.18^**^	0.13^**^	0.28^**^	0.16^**^	0.22^**^	0.12^**^
(4) (T2)	0.09^**^	0.14^**^	0.11^**^	0.21^**^	0.15^**^	0.27^**^	0.12^**^	0.24^**^
(5) (T1)	0.14^**^	0.11^**^	0.24^**^	0.14^**^	0.31^**^	0.18^**^	0.24^**^	0.16^**^
(5) (T2)	0.15^**^	0.17^**^	0.20^**^	0.17^**^	0.26^**^	0.34^**^	0.23^**^	0.28^**^

The convergent items (1)–(5) content could be found in Supplementary material. ^*^p < 0.01, ^**^p < 0.001. SSE, Scale of student egocentrism; SMCS, Student moral character scale; MCS, Moral competence scale.

#### 3.2.5 Discriminant validity

Regarding discriminant validity, results (Table 10) indicated that the total score of the CAS did not substantially correlate with four conceptually unrelated indicators across the two waves: Item (1) ( ∣r∣∈[0,0.10] ), item (2) ( ∣r∣∈[0.04,0.05] ), item (3) ( ∣r∣∈[0,0.09] ), and item (4) ( ∣r∣∈[0.05,0.09] ). The subscale score of the CAS also did not correlate with the four conceptually uncorrelated indicators across two waves: item (1) ( ∣r∣∈[0,0.08] ), item (2) ( ∣r∣∈[0.02,0.11] ), item (3) ( ∣r∣∈[0,0.09] ), and item (4) ( ∣r∣∈[0.01,0.14] ). A few correlation coefficients were significant at the 0.01 level, but the variance explained was low (Schumm et al., 1986). Therefore, Hypothesis 4a was supported. This provides evidence concerning four conceptually uncorrelated preference items, not evidence that the CAS is distinct from competing constructs such as multicultural attitudes.

**Table 10:** Correlation between the CAS with discriminant validity items.

Discriminant item	DC (T1)	DC (T2)	CD (T1)	CD (T2)	IA (T1)	IA (T2)	Acceptance total (T1)	Acceptance total (T2)
Discriminant items								
(1) (T1)	0.08^*^	0.05	0.07^*^	0.03	0.07^*^	0.03	0.10^**^	0.06
(1) (T2)	0.00	0.05	0.00	0.04	0.00	0.05	0.00	0.00
(2) (T1)	0.09^**^	0.11^**^	0.05	0.04	−0.02	−0.05	0.04	0.04
(2) (T2)	0.09^**^	0.09^**^	0.05	0.06	0.05	0.02	0.05	0.05
(3) (T1)	0.09^**^	0.01	0.08^*^	0.05	0.08	0.05	0.09^**^	0.09^**^
(3) (T2)	0.03	0.03	0.00	0.01	0.00	−0.02	0.00	0.00
(4) (T1)	0.05	0.02	0.09^*^	0.06	0.09^*^	0.05	0.09^**^	0.09^**^
(4) (T2)	0.01	0.08^*^	0.06	0.14^**^	0.06	0.16	0.05	0.05

^*^p < 0.01, ^**^p < 0.001. Discriminant items (1)–(4) content could be found in methodology.

However, the square roots of the AVE values for the three factors (0.68–0.73) were not greater than their inter-factor correlations (0.81–0.98). Hence, Hypothesis 4b was rejected (Table 6). In addition, the HTMT ratio suggested that only one factor pair (DC and IA) met the conventional discriminant validity criterion (i.e., HTMT_DC-IA_ = 0.82 < 0.85) (Henseler et al., 2015; Voorhees et al., 2016). Thus, Hypothesis 4c was rejected. Consequently, the correlation of conceptually unrelated indicators offered evidence for the total CAS, while the factor correlations and HTMT values raised concerns about the distinctiveness of the subscales.

#### 3.2.6 Reliability

Table 11 presents the reliability of the total CAS score and the three subscales. Cronbach’s α, CR, and McDonald’s ω were all >0.80. The MIIC ranged from 0.48 to 0.53 for the three subscales and the total scale ranged from 0.46 to 0.48 across both waves, respectively, indicating that items were sufficiently correlated to measure the same construct without being redundant. Overall, the CAS total scale and subscales demonstrated excellent internal consistency over time. Accordingly, Hypotheses 5a, 5b, and 5c were supported.

**Table 11:** Reliability of the CAS.

CAS	No. of items	Cronbach’s α	McDonald’s ω	CR	MIIC
Wave 1					
DC	5	0.84	0.84	0.83	0.52
CD	5	0.81	0.80	0.80	0.47
IA	10	0.90	0.88	0.88	0.48
Total	20	0.94	0.94		0.46
Wave 2					
DC	5	0.85	0.85	0.84	0.53
CD	5	0.83	0.82	0.82	0.49
IA	10	0.90	0.88	0.88	0.50
Total	20	0.94	0.94		0.48

## 4 Discussion

Study 2 supported a modified three-factor structure rather than the initially proposed four-factor structure. Although the four-factor model, comprising DC, CD, KR, and AO, was derived from the theory and consensus of the expert panel, CFA results showed that KR and AO could not be separated empirically. High correlation between KR and AO, together with a non-positive definite latent covariance matrix and problematic parameter estimates, indicated that the original four-factor model might over-differentiate different dimensions proposed. Hence, there is a need to re-visit the dimensions in the conceptual framework.

The revised three-factor solution can be interpreted in terms of behavioral, affective, and cognitive-evaluative aspects of acceptance. DC primarily reflects the behavioral aspect through engagement with people from different backgrounds, whereas CD primarily reflects the affective aspect through emotional comfort in such interactions. Likewise, IA primarily reflects the cognitive-evaluative aspect through recognizing others’ worth, appreciating differences, and accommodating diversity. Combining the dimensions of AO and KR into a single dimension is also theoretically meaningful, as both may represent related expressions of a broader cognitive orientation toward accepting difference (Walzer, 1997). Conceptually, they both involve accepting the legitimacy of differences and affirming the worth of others, although KR may additionally involve emotional restraint and inclusive conduct. Wang et al. (2022) also highlighted the interdependence between moral judgment and interpersonal conduct rather than treating them as entirely separate domains.

The final three-factor model retained several correlated item residuals sharing repeated wording or notions related to “different backgrounds,” “city,” “activities,” and “learning others’ opinions”. These residual correlations may indicate local dependence. In other words, these items share variance that is not adequately explained by the three latent factors. More specifically, we interpret this shared wording pattern as reflecting respect for people from different backgrounds regardless of context or behavior. In this sense, acceptance may involve contact, comfort, and appreciation.

Another strength of the CAS is its longitudinal invariance in large samples, confirming that the scale assesses the same construct across two time points. This is particularly relevant for university students, who are in the early stage of emerging adulthood, when acceptance of diversity is crucial (Gerson and Neilson, 2014). Emerging adulthood involves major shifts in worldview and life choices, therefore making it important to have a measure that maintains stability over time to track the development of acceptance in adolescents and adults. The present research design has 963 emerging adult participants, which has clear developmental and educational significance.

The non-significant association between the CAS total score and students’ egocentrism was unexpected. According to Golubickis et al. (2016), egocentrism may be related to self-perspective in the context of mindfulness, while egoism has also been examined in relation to broader personality characteristics (De Vries et al., 2009). However, these findings do not directly address acceptance of diversity. Therefore, the present finding should be interpreted cautiously. Further research is also needed to clarify the relationship between acceptance of diversity and egocentrism.

## 5 General discussion

This study developed and validated the CAS among university students in Hong Kong. Study 1 established the content validity of the initial CAS through the literature review, expert panel evaluation, and item refinement. These processes enabled the item pool to adequately serve as a culturally grounded instrument specifically tailored to Chinese university students, instead of a directly translated version of Western measures. Study 2 tested the factorial validity, factorial invariance, convergent validity, discriminant validity, and reliability of the CAS across two waves among Chinese university students in Hong Kong. To sum up, the CAS exhibited a stable factor structure and high internal consistency, and the evidence for convergent and discriminant validity was stronger for the total CAS than for its subscales. Taken together, the findings provide preliminary support for the CAS as a culturally grounded and psychometrically sound measure for assessing acceptance of diversity among Chinese university students.

This study has several unique contributions. The biggest contribution is the CAS is based on an integration of Western and Chinese understanding of acceptance within Chinese university context based on longitudinal data. Besides, the CAS items are aligned with the behavioral, affective and cognitive dimensions of acceptance. The scale draws on an orientation of contact of diversity while incorporating the relational-oriented responses associated with KR and AO. In particular, KR and AO extend the assessment of acceptance beyond contact with or tolerance of difference to include actively accepting, and supporting others (Chen, 2021). This broader framing reflects a relational orientation in which acceptance is not limited to interpersonal contact but also includes active efforts to promote inclusiveness and social cohesion. This emphasis on relational harmony and harmonious coexistence is also reflected in social service practices in Chinese communities, where multicultural competence incorporates relational harmony and cross-cultural adaptation (Wu et al., 2016).

The second contribution of this study is that it provides evidence for both the validity (content, factorial, convergent and discriminant validities) and reliability of the CAS, which strengthens its empirical foundation. Besides, by demonstrating longitudinal measurement invariance, the present study supports the potential use of the CAS for assessing changes in acceptance over time. With increasing openness to foreign talents in Hong Kong, China, it is important to equip future leaders with a strong acceptance of diversity. The CAS can be used in educational research, student development programs, diversity education, and leadership education. Its demonstrated longitudinal invariance provides preliminary support for the comparability of CAS scores across the two measurement occasions, which is a prerequisite for future research examining changes in acceptance over time. Zhu and Shek (2021) found that participation in a Service Leadership Course increased students’ life satisfaction, and similar programs may also foster attributes such as acceptance. The CAS can also be used as an outcome measure for positive youth development programs which attempt to enhance acceptance and empathy of young people (Tan et al., 2026; Zhu et al., 2025).

Although this study is pioneering, it has several limitations. First, it was conducted exclusively with Hong Kong university students. Given sociocultural differences between Hong Kong and Mainland China, such as divergent immigration policies, future research should examine CAS invariance across rural and urban regions in Mainland China to test its generalizability. Second, the study relied exclusively on students’ self-report data. Future research should incorporate perspectives from multiple informants to strengthen evidence of convergent validity. Third, the sample of this study only includes university students. Future research could assess measurement invariance among adolescents or emerging adults outside educational contexts. Fourth, there is a need to further ascertain the psychometric properties of the subscales of the CAS. Fifth, weak separation between the CAS factors also warrants caution in interpreting subscale scores, particularly between CD and IA. Future studies should compare alternative factor structures and examine whether the subscales predict relevant outcomes beyond what can be explained by the total CAS.

Sixth, the revised three-factor model and correlated residuals were informed by the same dataset used to assess their fit. This creates a risk of overfitting. Besides, the second wave involved the same participants and does not constitute an independent validation sample. The model should be tested in a new sample or in a prospectively defined development-and-validation design. Seventh, expert judgments alone cannot establish how well the item pool captures students’ lived experiences and culturally specific interpretations. Therefore, cognitive interviews and further qualitative validation with university students are needed in the future study.

## 6 Conclusion

In conclusion, the present study provided initial evidence to support the psychometric properties of the CAS among Chinese university students in Hong Kong. The CAS integrates relevant Western perspectives with Chinese understandings of acceptance of diversity, while recognizing both shared and differently emphasized aspects. This study examined content validity, factorial validity, measurement invariance, convergent validity, discriminant validity, and reliability. The research findings provide initial support for the CAS as a culturally grounded instrument for measuring diversity acceptance. Expert review and a two-wave study of Chinese university students in Hong Kong yielded preliminary evidence concerning its measurement properties. Despite limitations, the CAS offers a basis for further research on acceptance of diversity in university settings and for interventions designed to foster virtues among Chinese university students in Hong Kong.

## Data Availability

The raw data supporting the conclusions of this article will be made available by the authors, without undue reservation.
