# Supplementary material for: Development and validation of the Chinese Acceptance Scale for university students (CAS) in Hong Kong, China
Source: Front Psychol. 2026 Oct 14;17:1965526. doi: 10.3389/fpsyg.2026.1965526 (PMC13713284; doi:10.3389/fpsyg.2026.1965526)
Supplement: Supplementary file 1 [file Table_1.DOCX]

**Table S1**

*Items Used in the Convergent Validity Analysis*

| Traditional Chinese wording and English translation | | | | |
| --- | --- | --- | --- | --- |
| Morality | 1 | 你覺得你的道德是怎樣? |  |  |
|  |  | How do you rate your morality? | |  |
| Prosocial Attitude | 2 | 我關心社會上遭遇不幸的人 | |  |
|  |  | I care about people who have suffered from misfortune. | | |
|  | 3 | 如有機會，我會參加義務工作 | |  |
|  |  | I will participate in volunteer work if I have the chance. | | |
|  | 4 | 我同意每個人都應該受到法律的約束 | | |
|  |  | I agree that everyone should be subject to the law. | | |
|  | 5 | 如果我做錯事，我會感到羞恥 | |  |
|  |  | I would feel ashamed if I did something wrong. | | |

**Note.** Item 1 assessed self-rated morality and rated on a 5-point scale ranging from 1 (very low) to 5 (very high). Item 2-5 evaluated the prosocial attitude based on the Prosocial Attitude Scale. These items were measured using a 6-point Likert scale ranging from 1 (strongly disagree) to 6 (strongly agree).
